# The Feasibility of Japanese Histological Grade Classification for Predicting Renal Function Deterioration among Taiwanese Individuals with IgA Nephropathy

**DOI:** 10.3390/jcm12237339

**Published:** 2023-11-27

**Authors:** Cheng-Hsu Chen, Ming-Ju Wu, Shang-Feng Tsai

**Affiliations:** 1 Division of Nephrology, Department of Internal Medicine, Taichung Veterans General Hospital, Taichung 407219, Taiwan; cschen920@vghtc.gov.tw (C.-H.C.); wmj530@vghtc.gov.tw (M.-J.W.); 2 Department of Life Science, Tunghai University, Taichung 407224, Taiwan; 3 Department of Post-Baccalaureate Medicine, College of Medicine, National Chung Hsing University, Taichung 402204, Taiwan; 4 Ph.D. Program in Tissue Engineering and Regenerative Medicine, College of Medicine, National Chung Hsing University, Taichung 402204, Taiwan

**Keywords:** IgA nephropathy (IgAN), Japanese Histological Grade Classification (JHGC), composite renal outcome, end-stage kidney disease (ESKD), Oxford classification, glomerular filtration rate (GFR), proteinuria

## Abstract

Background: We aimed to validate the Japanese histological grading classification (JHGC) in our population of IgA immunoglobulin (IgAN) cases. Methods: We conducted a retrospective cohort study at Taichung Veterans General Hospital in Taiwan from January 2011 to December 2023. The process involved assessing JHGC’s clinical, histological, and merged grading system. Composite renal outcomes based on glomerular filtrate rate (eGFR) were considered. Results: The study included 359 IgAN by renal biopsies. Kidney function at the time of biopsy was suboptimal, with average SCr of 1.3 mg/dL, eGFR of 54.0 mL/min/1.732 m^2^, and urine protein–creatinine ratio (UPCR) of 1.2 mg/mg. JHGC effectively identified different severity levels of histological and clinical aspects in Taiwanese IgAN. Initial 4-histological classification showed significantly higher MEST-C scores (*p* < 0.001). Merging grade III and IV was reasonable in Japanese and Taiwanese populations. The clinical grading system (3C) was associated with histological status and proteinuria, but there was no significant trend with SCr, eGFR, and blood urea nitrogen. Significant differences were found among the three groups (log-rank *p* < 0.01), but C-grade I and II lacked significant difference in long-term renal outcomes. We separated UPCR < 0.5 mg/mg into two groups: eGFR≥ and <60 mL/min/1.732 m^2^. The new grading system effectively differentiated risk factors for renal outcomes (log-rank *p* < 0.01), suggesting the need for separation in Taiwanese IgAN. Conclusions: Our study externally validated JHGC in non-Japanese IgAN. Despite applicability to our population, we recommend a new classification specifically for Taiwanese IgAN patients with increased case numbers in eGFR ≥ 60 mL/min/1.732 m^2^ and UPCR < 0.5 g/day group.

## 1. Introduction

Immunoglobulin A nephropathy (IgAN) is the most common primary glomerular disease [1], particularly prevalent in East Asia [1,2,3], especially Japan. Despite its previously considered benign nature, IgAN’s course has proven otherwise [4,5,6,7,8]. Despite extensive research, the underlying pathogenesis of IgAN remains elusive, resulting in the absence of a definitive treatment. Moreover, variations in clinical manifestations, mechanisms, and outcomes across different countries and races [9] pose challenges to the generalization of predictive models. Numerous predictive models [4,6,7,8], incorporating clinical, laboratory, and histological variables, have been reported for renal outcomes. Furthermore, guidelines for IgAN management differ between Japanese [10] and non-Japanese groups [11], with Japan implementing regular urinary analysis in elementary school and tonsillectomy, supported by local evidence [12,13,14], for renal benefits. For IgAN, predictive models matter significantly. Inaccurate classification could potentially mislead both physicians and patients, leading to an incorrect assessment of the severity of renal injury in individuals with IgA nephropathy. Consequently, this misinterpretation may result in the implementation of continuous and inappropriate therapy for these patients.

The Oxford classification (MEST-C for mesangial hypercellularity, endocapillary hypercellularity, segmental glomerulosclerosis, tubular atrophy/interstitial fibrosis, and crescents) has been the most widely utilized histological classification globally since 2009 [15,16]. Developed by the International IgA Nephropathy Network in collaboration with the Renal Pathology Society, it is based on clinical data and kidney biopsies from 265 white patients and East Asian patients who were followed for a median of five years [15,16]. Many institutes adopt the Oxford classification for histological analysis, leading to the development of predictive models for renal function based on this system. However, only 20 adult Japanese patients were included in the development of classification [15]. Additionally, the Oxford classification, which predicts renal function solely based on histological variables, highlights the necessity for improved prognostic models. Therefore, since 2012, the Japan Society of Nephrology has employed the unique Japanese Histological Grade Classification (JHGC) to predict renal outcomes [17,18,19]. This JHGC was proposed by the Special IgAN Study Group of the Progressive Glomerular Diseases Study Committee, organized by the Ministry of Health, Labor and Welfare of Japan. This grading classification relies on clinical parameters (proteinuria and glomerular filtration rate (GFR)) and histological variables. JHGC was validated in 2015 in a Japanese center with good correlation to renal outcome [20]. Despite being widely employed for over a decade in Japan [20,21], JHGC lacks external review for the feasibility in other races and countries [21]. In Taiwan, being part of East Asia, we are curious about the predictive power of JHGC and its applicability to Taiwanese individuals. Therefore, this study aims to validate the JHGC in the Taiwanese population with IgAN while exploring novel predictive systems.

In a prior study [22], we validated the Haas classification of IgAN in our cohort, demonstrating its applicability in predicting renal outcomes for the Taiwanese population. The previous study was based on our retrospective cohort between January 2003 and December 2013. Since 1980, our institute has accumulated over 8000 instances of renal biopsy, providing a well-documented IgAN cohort with comprehensive clinical, laboratory, and histological data. Additionally, long-term follow-up data on kidney function further bolster the study’s foundation. Consequently, we conducted this study with a much longer duration of follow-up to externally validate the JHGC in our IgAN cohort, thereby contributing valuable insights to the field.

## 2. Materials and Methods

### 2.1. Definition of Population

Between January 2011 and December 2023, we conducted a retrospective cohort study at Taichung Veterans General Hospital in Taiwan. The study included participants who were aged ≥ 20 years old and had undergone their first native renal biopsy with a diagnosis of IgAN at our medical center. The study received approval from the institutional review committee (approval number: CE15125B). This study was conducted according to established ethical guidelines in our institute and the informed consent was waived by the ethics committee because of de-identified data.

### 2.2. Other Data Collection

Data for this study were obtained by reviewing medical records from the cohort. Baseline information, such as gender, age (years old, y/o), and body mass index (BMI) (kg/m^2^) were collected at the time of each patient’s renal biopsy. Additionally, data from blood samples were gathered, which included measurements for blood urea nitrogen (BUN) (mg/dL), serum creatinine (SCr) (mg/dL), and estimated GFR (calculated using the Modification of Diet in Renal Disease equation) (mL/min1.732 m^2^). The analysis also considered white blood cell count (/cumm), hemoglobin (g/dL), platelet count (/cumm), metabolic profile (total cholesterol (TC) (mg/dL), triglyceride (mg/dL), low-density lipoprotein (LDL) (mg/dL), high-density lipoprotein (HDL) (mg/dL), fasting blood sugar (FBS) (mg/dL), glycated hemoglobin (HbA1c) (%), and uric acid (UA) level (mg/dL)), and albumin (g/dL). The study took into account various immunological markers, including complement 3 (C3), C4, IgG, IgM, IgA, and IgE. Urine samples were also analyzed for spot urine protein–creatinine ratio (mg/mg) (UPCR), hematuria, and pyuria. Pathologists conducted a comprehensive analysis of all pathological samples, and all enrolled participants were diagnosed with IgAN based on the Oxford classification (MEST-C). 

### 2.3. Outcome Definitions

Renal failure was defined in accordance with the international consensus definitions of clinical trial outcomes for kidney failure (2020) [23]. We defined renal outcome according to the above study, including clinical criteria such as receipt of a kidney transplant, dialysis performed for at least 4 weeks, participant death where kidney replacement therapy was never initiated, and advanced chronic kidney disease as the underlying cause of death. Additionally, we also included eGFR-based outcomes, including doubling of SCr, percent decline in eGFR of ≥50% from a baseline start point sustained over at least 4 weeks, and eGFR < 15 mL/min/1.73 m^2^ sustained over at least 4 weeks. The aforementioned outcomes were gathered from medical records within our institute. The data were independently recorded by two nurses and subsequently verified by a single nephrologist to ensure accuracy.

### 2.4. Process of Taiwanese IgAN in Japanese Histological Grade Classification (JHGC)

The detailed JHGC classification was as below [17,19]. The JHGC classification is a merged system with 4 grades: low, moderate, high, and very high risks, based on 3 histological (3H) and 3 clinical grades (3C). The 3 clinical classifications (3C) are as follows: C-grade I (<0.5 g/day of proteinuria), C-grade II (≥0.5 g/day of proteinuria and eGFR ≥ 60 mL/min/1.732 m^2^), and C-grade III (≥0.5 g/day of proteinuria and eGFR < 60 mL/min/1.732 m^2^). Regarding the histological classifications, initially, there were 4 grades (4H) (H-grades I, II, III, and IV), which were based on the percentage of glomeruli with pathological variables (cellular crescent, tuft necrosis, fibrocellular crescent, global sclerosis, segment sclerosis, and fibrous crescent). However, H-grades III and IV were later merged into a new grade (grade III + IV). Consequently, the final histological classification (3H) consists of three grades: H-grades I, II, and III + IV. Subsequently, the 3H and 3C classifications were multiplied to form 9 groups, which were then merged into 4 groups (low, moderate, high, and very high risks). Therefore, we validated Taiwanese IgAN following the thinking process of JHGC.

**Figure 1:**
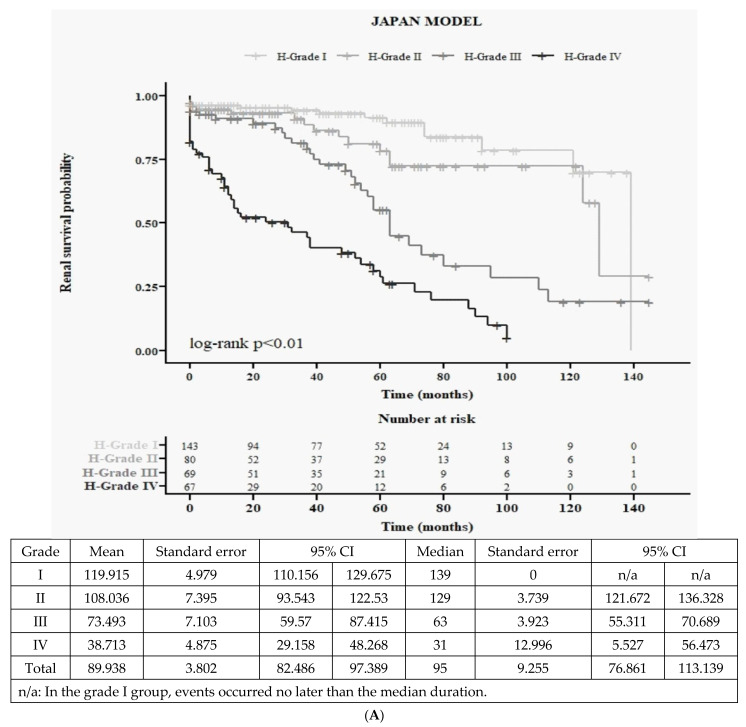

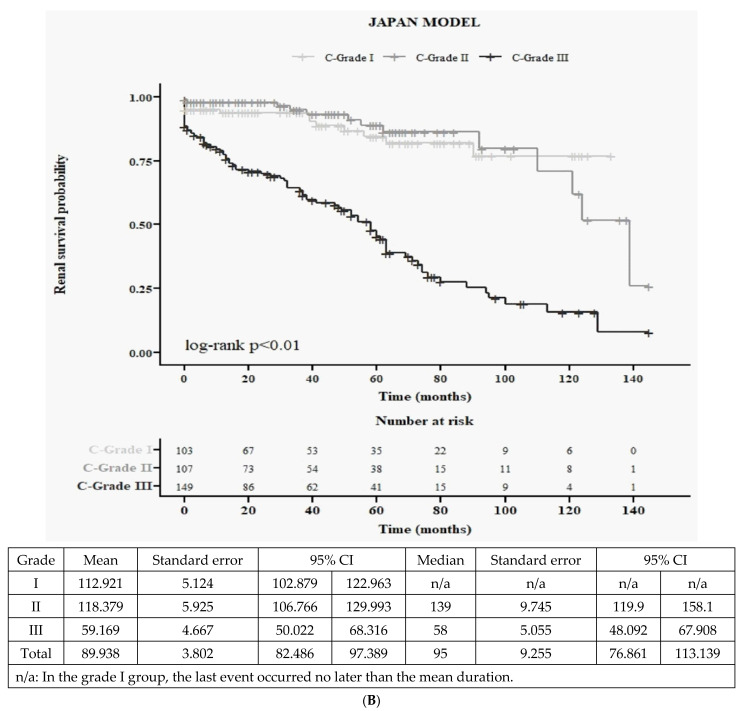

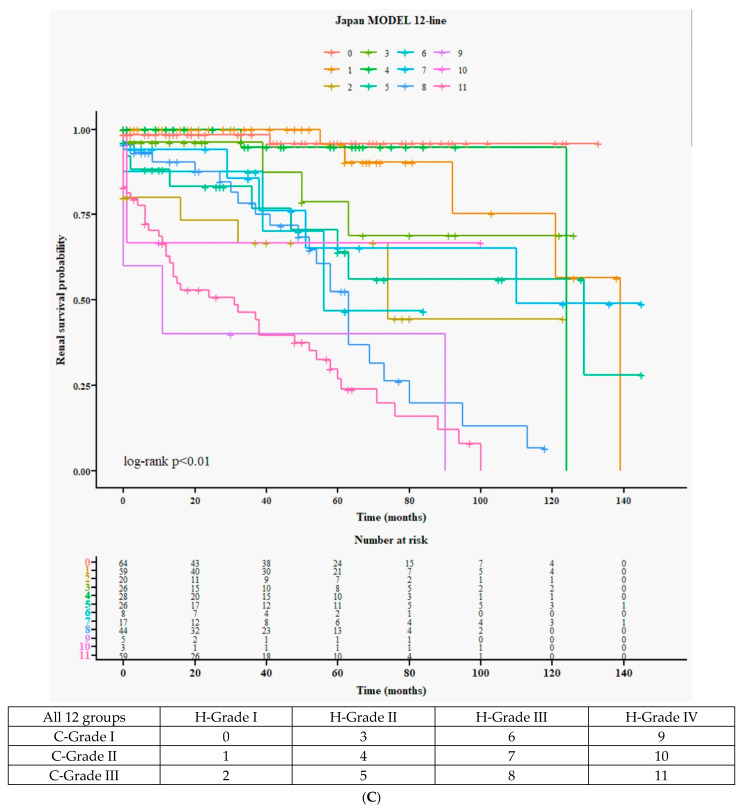

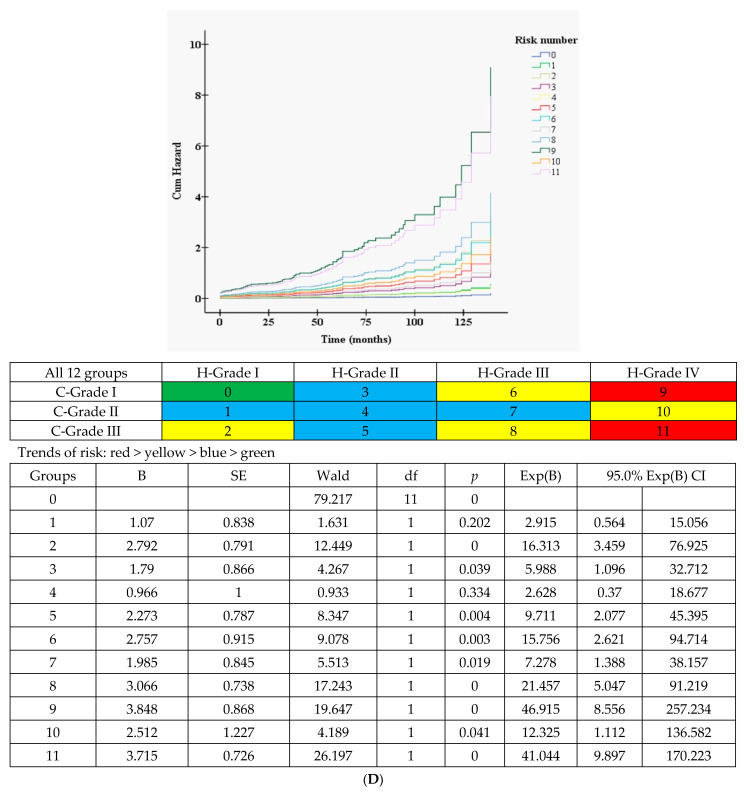

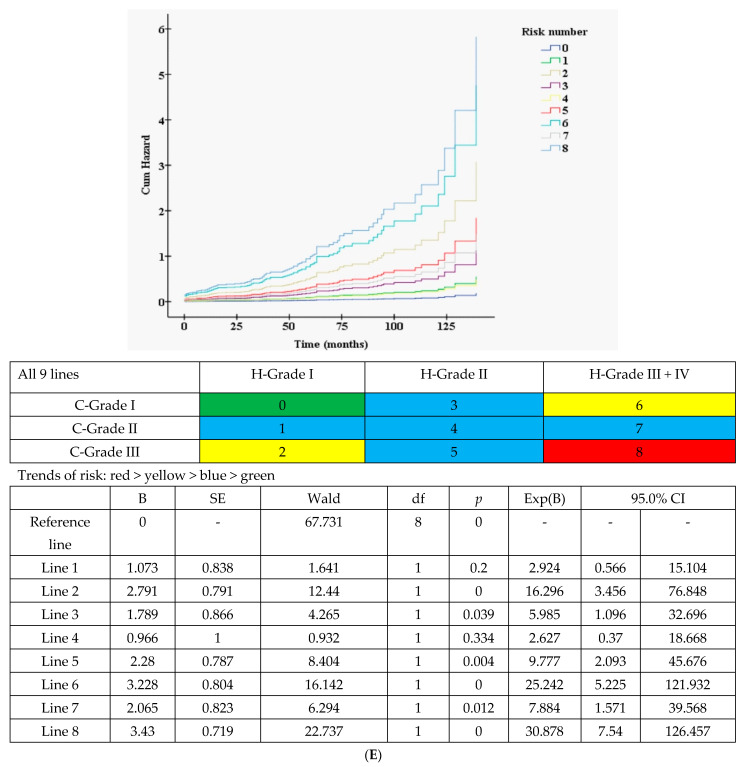

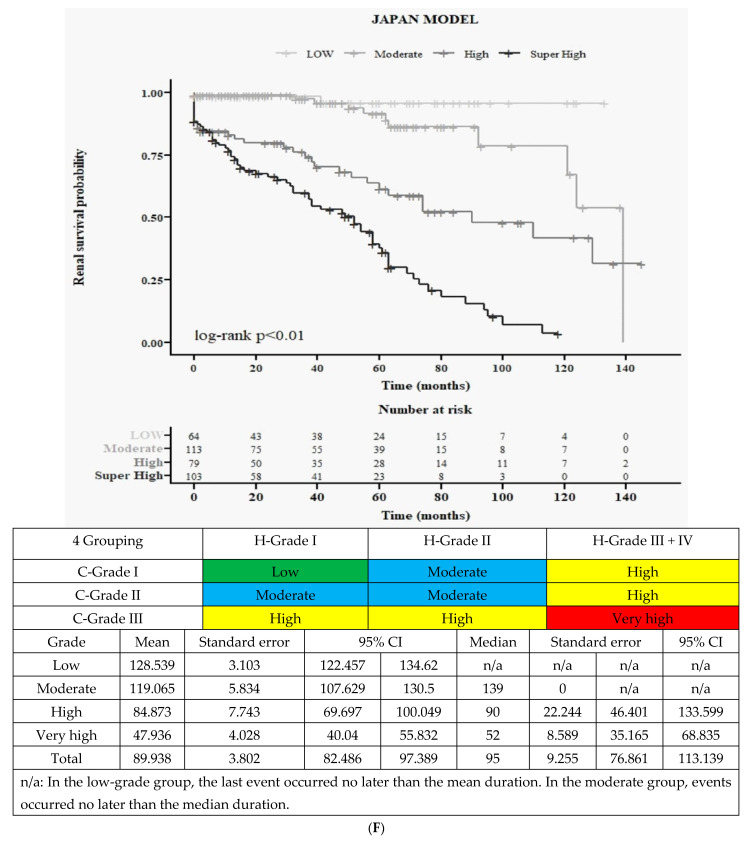
Kaplan–Meier curves for renal outcome of Taiwan population based on Japanese Histological Grade Classification (JHGC). (**A**) Histological classifications (4H). (**B**). Clinical classification (3C). (**C**) All 12 survival curves based on 4H × 3C (4 histological grades × 3 clinical grades). (**D**) Cox accumulative hazard curve among all 12 groups based on 4H × 3C (4 histological grades × 3 clinical grades). (**E**). Cox accumulative hazard curve among all 9 groups based on merged classification (3H × 3C) (merged H-grades III and IV, then 3 histological grades × 3 clinical grades). (**F**). Merged classification (incorporated 9 groups into 4 groups as final JHGC system).

We verified the JHGC classification step by step to investigate its predictive power in Taiwanese IgAN (Supplementary Figure S1). For every step of group or grades, we made Kaplan–Meier curves to see the predictive power. Firstly, we showed the baseline data of Taiwanese IgAN according to 4 grades of JHGC (Table 1). For more detail, all baseline data were also shown according to 4H (Supplementary Table S1) and 3C (Supplementary Table S2). We drew Kaplan–Meier curves for 4H (Figure 1A) and 3C (Figure 1B), and then we multiplied 4H and 3C as 12 groups. The 12 Kaplan–Meier curves are shown in Figure 1C. After the merged H-grades III and IV, there were 9 curves (Figure 1D), which were multiplied from 3C and 3H. Finally, the 9 curves were merged into 4 grades (Figure 1E).

We explored an alternative grading system for clinical conditions to assess any improvement in predictive power in Taiwanese IgAN. The new system, known as new 4 clinical classification (new 4C), is as follows: C-grades I (eGFR ≥ 60 mL/min/1.732 m^2^ and UPCR < 0.5 mg/mg), II (eGFR ≥ 60 mL/min/1.732 m^2^ and UPCR ≥ 0.5 mg/mg), III (eGFR< 60 mL/min/1.732 m^2^ and UPCR < 0.5 mg/mg), and IV (eGFR < 60 mL/min/1.732 m^2^ and UPCR ≥ 0.5 mg/mg).

**Figure 2:**
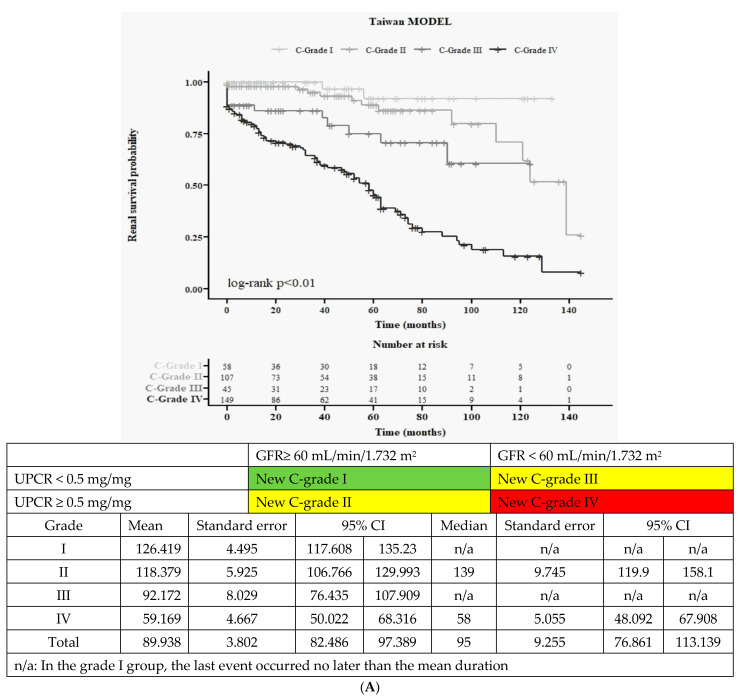

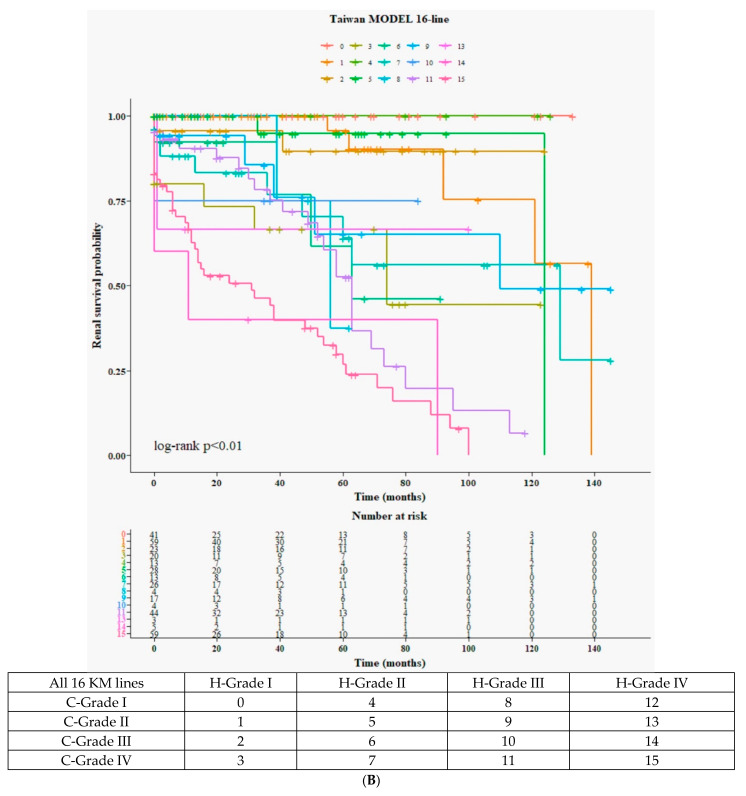

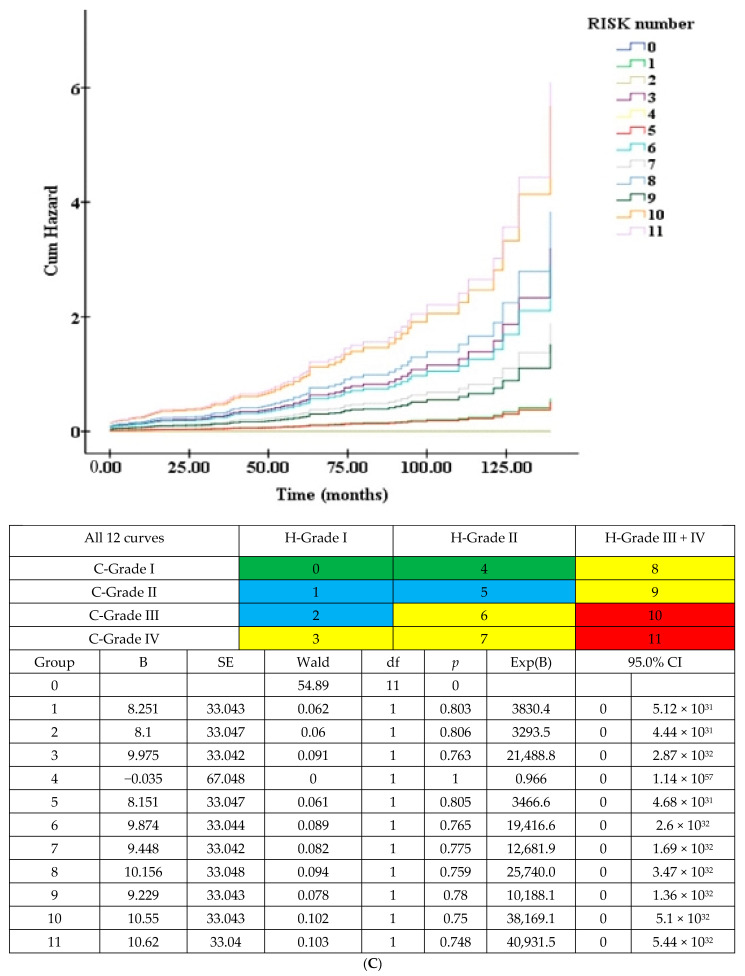

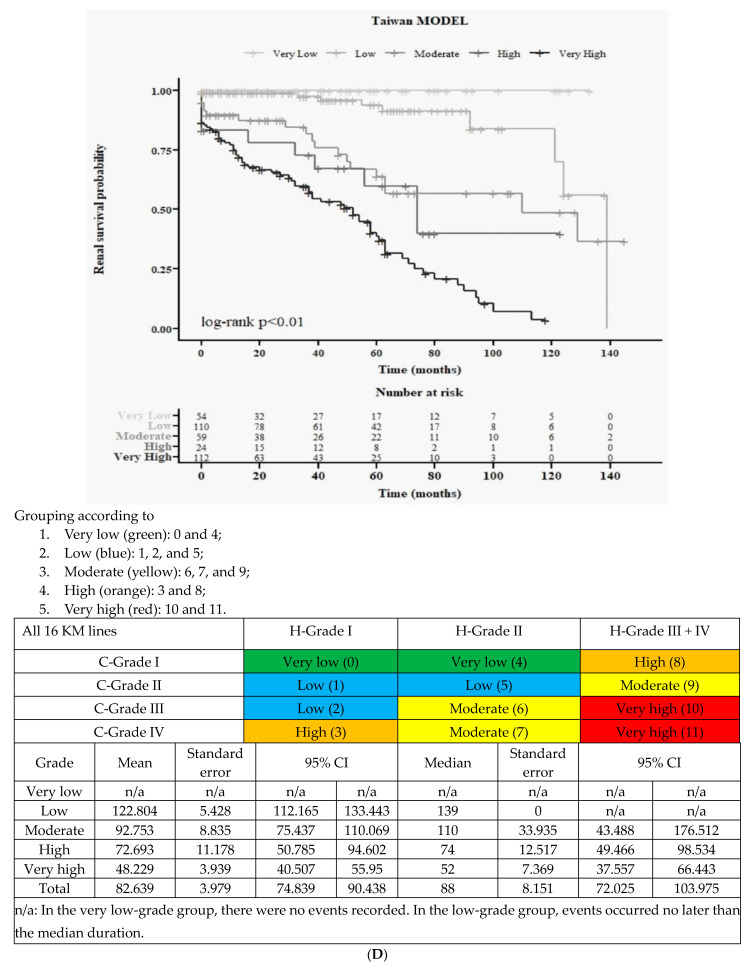
Kaplan–Meier curves for renal outcome of Taiwan population based on new classification model. (**A**). New adjusted clinical classification (new 4C). (**B**). All 16 survival curves based on merged classification (4H × new 4C). (**C**). Cox accumulative hazard curve among all 12 groups based on merged classification (3H × new 4C). (**D**). Merged classification (12 groups incorporated into 5 groups).

The baseline data for this new 4 clinical classification (new 4C) can be found in Supplementary Table S3, while the corresponding Kaplan–Meier curves are presented in Figure 2A. Subsequently, we obtained 16 curves (Figure 2B) by multiplying 4H and new 4C. Similarly to the JHGC system, we also merged H-grades III and IV into a new grade: 4H to 3H. Consequently, we derived 12 curves (Figure 2C) by combining new 4C and 3H. Finally, all 12 curves were consolidated into five grades (Figure 2D) based on the risk of composite renal outcomes, resulting in the following categories: very low, low, moderate, high, and very high.

### 2.5. Statistical Analyses

In this study, continuous variables were presented as the median (Q1, Q3), providing a comprehensive understanding of the central tendency and distribution of the data. Categorical data were illustrated using numbers (percentages) to represent the frequency and proportion of different categories. To assess normality, the Kolmogorov–Smirnov test was employed. For comparisons of categorical variables, the chi-square test was used, while descriptive statistics, the Kruskal–Wallis test, and the chi-square test were utilized when appropriate. The statistical significance level was set at a *p*-value of less than 0.05.

For survival analysis, the Kaplan–Meier method was employed to generate renal survival curves. The log-rank test was used to compare the renal survival rates among various groups with different risk factors. Variables that showed statistical significance in the univariate analysis were included in an accumulative hazard risk (HR) curve for the risk of composite renal outcomes. The assumption of proportional hazards was evaluated using scaled Schoenfeld residuals. Hazard ratios and their corresponding 95% confidence intervals (CI) were reported. We utilized the aforementioned statistical methods for risk reclassification to improve the predictive capability of composite real outcomes.

We did not choose Harrell’s C statistic for the following reasons. Firstly, the JHGC model development and subsequent validations primarily relied on ROC curves and Kaplan–Meier survival curves, omitting the use of Harrell’s C statistic. Secondly, Harrell’s C statistic only provides a general idea about a model. As a single number, it summarizes the discrimination of a model but does not communicate all the information ROC plots contain and lacks direct clinical application. Finally, the C statistic is only a measure of discrimination, not calibration, so it provides no information regarding whether the overall magnitude of risk is predicted accurately. 

Statistical analysis was conducted using SPSS software, version 22 (SPSS Inc., Chicago, IL, USA), and R software, version 4.3.1. A two-tailed *p*-value of less than 0.05 was considered statistically significant.

## 3. Results

The complete process of verification of JHGC in Taiwanese can be reviewed based on Supplementary Figure S1.

### 3.1. Baseline Characteristics of Taiwanese IgAN Cohort

Between January 2011 and December 2023, a total of 359 renal biopsies confirmed IgAN (Table 1). The patients were relatively young, with an average age of 46 years, and 52.4% of them were male. Moreover, they exhibited relatively low prevalence of metabolic syndrome, including an average BMI of 23.5 kg/m^2^, FBG levels of 94 mg/dL, HbA1c levels of 5.5, TC levels of 187.5 mg/dL, triglyceride levels of 122 mg/dL, LDL levels of 110.5 mg/dL, and UA levels of 6.6 mg/dL. 

As for renal function, the patients’ kidney function was not optimal at the time of renal biopsy, with an average SCr level of 1.3 mg/dL, eGFR of 54.0 mL/min/1.732 m^2^, UPCR of 1.2 mg/mg, and 86.4% of patients having hematuria. Regarding the pathological status, the distribution of lesions was as follows: 58.1% with M1, 29.6% with E, 62.4% with S, 20.5% with T1, and 10.7% with C1. 

### 3.2. Baseline Characteristics According to the Japanese Histological Grade Classification (JHGC)

#### 3.2.1. Merged Japanese Histological and Clinical Grading System (Merged 3H and 3C) 

We separated the entire Taiwanese population according to JHGC (Table 1). With increasing severity of JHGC, patients were found to be significantly older (*p* < 0.001) and have lower hemoglobin (*p* < 0.001), lower platelet counts (*p* = 0.001), lower serum albumin (*p* < 0.001), higher triglyceride (*p* = 0.003), higher BUN (*p* < 0.001), higher SCr (*p* < 0.001), lower eGFR (*p* < 0.001), higher UA (*p* < 0.001), lower C3 (*p* = 0.008), higher C4 (*p* < 0.001), lower IgM (*p* < 0.001), higher UPCR (*p* < 0.001), more M1 (*p* < 0.001), more E (*p* < 0.001), more T1 (*p* < 0.001), more T2 (*p* < 0.001), and more C1 histological lesions (*p* = 0.046). In general, this JHGC can effectively identify the different severity of histological and clinical aspects in Taiwanese IgAN.

#### 3.2.2. Japanese Histological Grading System (4H): Baseline Characteristics and Kaplan–Meier Survival Curves 

We separated the entire Taiwanese population according to the histological grading system of JHGC (4H, Supplementary Table S1). As the histological grade increased, patients had significantly lower hemoglobin (*p* < 0.001), lower albumin (*p* < 0.001), higher triglyceride (*p* = 0.002), lower HDL (*p* < 0.001), higher SCr (*p* < 0.001), lower eGFR (*p* < 0.001), higher UA (*p* < 0.001), higher C4 (*p* < 0.001), lower IgM (*p* = 0.001), and higher UPCR (*p* < 0.001). This histological grading system of JHGC is correlated with clinical conditions. Furthermore, concerning the Oxford classification, this histological grading system also showed significantly higher MEST-C scores in almost all MEST-C (*p* < 0.001). It is evident that this histological grading system of JHGC is consistent with the Oxford MEST-C classification. However, these differences in MEST-C did not exist between grades III and IV, with percentages of M being 74.6% for grade III and 72.3% for grade IV, percentages of E being 38.8% for grade III and 46.2% for grade IV, percentages of S being 85.1% for grade III and 84.6% for grade IV, and percentages of T1 and C1 being 37.3% for grade III and 46.2% for grade IV, respectively. This finding further supports the notion that merging grades III and IV is a reasonable approach not only in Japanese but also in Taiwanese populations. For composite renal survival (Figure 1A), there is statistical significance among all four groups according to the 4H classification (log-rank *p* < 0.01).

#### 3.2.3. Japanese Clinical Grading System (3C): Baseline Characteristics and Kaplan–Meier Survival Curves

We classified the entire Taiwanese population according to the clinical grading system of JHGC (3C, Supplementary Table S2). As the clinical grading of JHGC worsened, IgAN patients showed significant differences in various parameters. Specifically, they had lower hemoglobin (*p* < 0.001), lower albumin (*p* < 0.001), higher TC (*p* < 0.001), and higher LDL (*p* = 0.01). However, even with statistical significance among three groups, there was no trend according to worse clinical grading (3C) over the following variables: age (*p* < 0.001), platelet count (*p* < 0.001), UA (*p* < 0.001), C3 (*p* = 0.001), C4 (*p* = 0.004), IgG (*p* = 0.02), and IgM (*p* = 0.012).

The clinical grading system of JHGC showed a correlation with the histological findings of the Oxford classification. As the clinical grading worsened, patients displayed more M1 (*p* < 0.001), E (*p* < 0.001), S (*p* < 0.001), T1 (*p* < 0.001), T2 (*p* < 0.001), and C2 (*p* = 0.014). In addition, in the later stages, patients exhibited significantly higher UPCR (236 vs. 1510 vs. 1934, *p* < 0.001). However, while there was statistical significance among all three groups (all *p* < 0.001), the SCr, eGFR, and BUN did not follow a worsening trend based on the higher grade.

Overall, this clinical grading system (3C) of JHGC was associated with different severities of histological status and proteinuria. However, there was no trend observed with worse SCr, eGFR, and BUN.

Similarly, for renal survival (Figure 1B), there is statistical significance among all three groups according to the 3C classification (log-rank *p* < 0.01). However, it is evident that C-grades I and II did not exhibit a significant difference in long-term composite renal outcome. Therefore, in the Taiwanese IgAN cohort, there may be a need to reorganize the clinical grading system to improve the predictive power for composite renal outcomes.

### 3.3. Renal Outcome According to Various Grading Groups

#### 3.3.1. Detailed 12 Survival Curves (Figure 1C) and Cox Accumulative HR (Figure 1D) from 4H × 3C

All 12 groups resulting from 4H × 3C were presented with Kaplan–Meier survival curves (Figure 1C) and Cox accumulative HR (Figure 1D). The general trend is consistent, showing that higher clinical stage and histological stage correspond to worse renal outcomes, with the worst outcome in group no. 11 and the best in group no. 0. Within each C-grade, worse H-grade is associated with worse renal outcomes. Specifically, all C-grade II (group nos. 1, 4, 7, and 10) performed better than all C-grade III (group nos. 2, 5, 8, and 11) in each H-grade category, respectively. This suggests that under UPC ≥ 0.5 mg/mg, within each histological grade, eGFR < 60 mL/min/1.732 m^2^ is associated with worse outcomes than eGFR ≥ 60 mL/min/1.732 m^2^.

However, it is important to note that the risk of group no. 3 is similar to that of group nos. 4 or no. 5. Additionally, the risk of group no. 6 is similar to that of group no. 8, and the risk of group no. 9 is similar to that of group no. 11. These findings may suggest that C-grade I needs to be further subdivided into more detailed groups in the Taiwanese IgAN.

#### 3.3.2. Nine Curves According to 3H (merged H-grade III + IV) × 3C (Figure 1E)

All nine groups resulting from 3H × 3C were presented with Cox accumulative HR (Figure 1E). The general trend is consistent, indicating that higher clinical stage and histological stage are associated with worse renal outcomes, with the worst outcome observed in group no. 8 and the best in group no. 0. However, it is worth noting that the risk of group no. 6 is worse than that of group no. 7. This finding suggests that group no. 6 may need to be further subdivided into more detailed categories in the context of Taiwanese IgAN.

#### 3.3.3. Final All Four Groups According to Merged 3H and 3C (Figure 1F)

We grouped Taiwanese IgAN into four groups according to final JHGC. Generally, this classification system can differentiate different risks of renal outcomes.

### 3.4. New Clinical Grading System (New 4C): Baseline Characteristics and Kaplan–Meier Survival Curves

Based on the findings of Figure 1B,D,E, where C-grades I and II did not show a significant difference in long-term composite renal outcome, we decided to further separate C-grade I (UPCR < 0.5 mg/mg) into two grades. As a result, we now have four grades in clinical stages (new 4C): C-grades I (eGFR ≥ 60 mL/min/1.732 m^2^ and UPCR < 0.5 mg/mg), II (eGFR ≥ 60 mL/min/1.732 m^2^ and UPCR ≥ 0.5 mg/mg), III (eGFR < 60 mL/min/1.732 m^2^ and UPCR < 0.5 mmg/mg), and IV (eGFR < 60 mL/min/1.732 m^2^ and UPCR ≥ 0.5 mg/mg). We will now proceed to validate the predictive power for renal outcomes in this Taiwanese IgAN cohort using this updated clinical grading system.

#### 3.4.1. Baseline Characteristics (Supplementary Table S3)

In Supplementary Table S3, patients with worse new C-grade were found to have significantly older age (*p* < 0.001), lower hemoglobin (*p* < 0.001), higher FBG (*p* = 0.049), higher TC (*p* = 0.001), higher BUN (*p* < 0.001), higher SCr (*p* < 0.001), lower eGFR (*p* < 0.001), and lower C3 (*p* < 0.001). Moreover, for all MEST scores, they were more severe in individuals with worse new clinical grading (all *p* < 0.001).

#### 3.4.2. Kaplan–Meier Survival Curves for Renal Function (Figure 2A)

The renal survival curves based on the new C-grading system are shown in Figure 2A. This updated C-grading system effectively differentiates various renal outcomes with statistical significance (log-rank < 0.01).

### 3.5. Renal Outcome Prediction According to Various Adjusted Grading Groups

#### 3.5.1. Detailed 16 Curves from 4H × 4C (Figure 2B)

We then validated renal outcomes using the four histological and the new clinical grading system (4H × new 4C) (Figure 2B). However, due to limited case numbers, we were unable to observe a clear trend in renal survival.

#### 3.5.2. Twelve Curves According to 3H (Merged H-Grade III + IV) × New 4C (Figure 2C)

We utilized the three histological grades and the new clinical grading system (3H × new 4C) to predict renal function (Figure 2C). The general trends are consistent, showing that worse H-grade and C-grade correspond to worse renal outcomes: the highest risk is in group no. 11, and the lowest risk is in group no. 0. Within each H-grade, a worse C-grade is associated with higher risks, and similarly, within each C-grade, a worse H-grade is associated with higher risk.

#### 3.5.3. Final All Five Groups According to Merged 3H and New 4C (Figure 2D)

Finally, we grouped all 12 lines into five groups according to the risk: very low, low, moderate, high, and very high groups (Figure 2D and Table 2). The baseline characteristics are presented in Table 2. When patients had a higher new adjusted grade, they exhibited lower hemoglobin (*p* < 0.001), lower albumin (*p* < 0.001), higher triglyceride (*p* < 0.001), higher BUN (*p* < 0.001), higher SCr (*p* < 0.001), lower eGFR (*p* < 0.001), lower IgM (*p* = 0.001), and higher UPCR (*p* < 0.001). Additionally, regarding MEST-C, they had more M1 (*p* < 0.001), more E (*p* < 0.001), and more T2 (*p* < 0.001).

In this new grading system, the five groups effectively differentiate different risk factors for renal outcomes (log-rank *p* < 0.01) (Figure 2D). Compared to JHGC (Figure 1F), it appears that both systems can be used in Taiwanese IgAN. However, we further separated the previous C-grade I (UPCR < 0.5 mg/mg) into new C-grades I (UPCR < 0.5 mg/mg and eGFR ≥ 60 mL/min/1.732 m^2^) and III (UPCR < 0.5 mg/mg and eGFR < 60 mL/min/1.732 m^2^) (Figure 2C): no. 0 (very low) vs. no. 2 (low), no. 4 (very low) vs. no. 6 (moderate), and no. 8 (high) vs. no. 10 (very high). In Taiwanese IgAN, these two groups should be separated due to apparently different risks.

## 4. Discussion

In this study, we are the first to conduct an external review of the feasibility of JHGC in a non-Japanese population. The final JHGC (four grades) can be used in the Taiwanese population to predict composite renal outcome (Figure 1F). However, we have expanded the three clinical grades to four new clinical stages (Figure 2D). This new grading system can better differentiate Taiwanese IgAN patients, especially those with UPCR < 0.5 mg/mg and eGFR < 60 or ≥60 mL/min/1.732 m^2^.

For histological classification, the major difference between JHGC and the globally used Oxford classification [24] is the glomerular lesion percentage score (GLPS), which was assessed in each patient and categorized into histologic grades. However, while JHGC uses a lumped system, the Oxford classification provides a more detailed classification system based on MEST-C criteria [24]. From our study, we considered that this GLPS can also be used in our population. Firstly, the correlation of histological classification in JHGC in Taiwanese IgAN also shows a significant correlation with all MEST-C scores and clinical outcomes (all *p* < 0.001 in SCr, eGFR, and UPCR) (Supplementary Table S1). Additionally, the pathognomonic finding for IgAN is the presence of mesangial deposits of IgA in the mesangial area of glomeruli. Therefore, in our population, we believe that using this GLPS as the histological classification for renal outcome prediction is still appropriate.

There are many studies [25,26,27,28,29,30,31] aimed at predicting renal outcomes using either clinical data (proteinuria, hypertension, hematuria, SCr, eGFR, or proteinuria) or histological data (MEST-C or glomerular lesion percentage score (GLPS)). Interestingly, in a study with a 2-year follow-up conducted by the Oxford Derivation, North American Validation, and VALIGA Consortia [32], they did not find any significant improvement in prediction by adding MEST to clinical data (eGFR, proteinuria, and mean arterial blood pressure). In addition, from a review article in 2018 [33], there is no available risk prediction model using clinical and histological predictors that has been sufficiently validated for routine use. However, in the JHGC system since 2012 [19], they were the first to combine clinical and histological grading. This combination resulted in increased accuracy in predicting ESKD in Japanese IgAN patients. Moreover, another Asian population study in 2019 also showed that the best predictive model was combining clinical and histological data [34]. Similarly, in this Taiwanese IgAN study, the combined system (Figure 1F) demonstrated a higher predictive power than either histological (Figure 1A) or clinical classification (Figure 1B).

The reduction of eGFR at diagnosis Is associated with a worse kidney prognosis in many previous studies [4,5,35,36,37,38,39,40,41]. In our new classifications (Figure 2D) (new 4C, Figure 2A), we observe distinct clinical outcomes between new C-grade I (eGFR ≥ 60 mL/min/1.732 m^2^, UPCR < 0.5 g/day) and new C-grade III (eGFR < 60 mL/min/1.732 m^2^, UPCR < 0.5 g/day). Although the original JHGC (Figure 1F) can be used in our population, in Supplementary Table S2, there was no trend observed with worse SCr, eGFR, and BUN. Similarly, in Figure 1B, even though there is statistical significance among all three groups according to the 3C classification (log-rank *p* < 0.01), it is evident that C-grades I and II did not exhibit a significant difference in long-term composite renal outcome. Thus, our new grading system better differentiates these two groups. In the JHGC system [19], the reason for combining these two groups initially was that there were only seven cases included with eGFR ≥ 60 mL/min/1.732 m^2^ and UPCR < 0.5 g/day, and no cases resulted in renal death. Furthermore, in the multivariate logistic analysis from their cohort [19], proteinuria and eGFR were identified as significantly independent variables [19]. The area under the curve for proteinuria and eGFR was 0.774 and 0.777, respectively. Hence, they combined these two groups [19,21]. In our study, we expanded the number of cases (n = 58) in the new C-grade I category (eGFR≥ 60 mL/min/1.732 m^2^ and UPCR < 0.5 g/day). In our final analysis (Figure 2C), the renal outcomes were significantly different in these two groups: group 0 vs. 2, 4 vs. 6, and 8 vs. 10. Therefore, compared to the JHGC [19], we provided a much larger number of cases in the eGFR ≥ 60 mL/min/1.732 m^2^ and UPCR < 0.5 g/day group (n = 58 vs. 7), and we suggest that we cannot combine new C-grades I and III due to their different renal outcomes. Our data are consistent with the latest analysis from JHGC [42], which was published just 7 months ago. The secondary analysis from a nationwide multicenter study in Japan [43] was also recently published. They found that after a comparison with JHGC data, the overall HR is 9.67 between the two groups [42]. However, they have not yet revised the final JHGC data merged from clinical and new clinical grades. In this study, we are the first to provide a new grading system, merging histological and new clinical grading systems.

There are some limitations to this study. Firstly, the treatment methods varied among the groups with different risks, and they were not taken into consideration for outcome prediction. Secondly, over time, the availability of medications for IgAN, such as renin–angiotensin system inhibitor and sodium–glucose cotransporter 2 inhibitors, has increased, and these treatments were not accounted for in our analysis. Thirdly, we did not have any cases in our study population that underwent tonsillectomies, which further adds to the divergence in treatment conditions between Taiwanese and Japanese groups. Fourthly, this study is a retrospective observational study and we cannot demonstrate direct cause and effect risk associations and it is possibly affected by residual confounding. Finally, the renal outcome of JHGC is end-stage kidney disease (ESKD). For our study, our renal outcome is composite renal outcome. Despite the above limitations, we still found that JHGC could be effectively applied to non-Japanese IgAN with only a few adjustments. Conclusions

JHGC was the first to predict renal outcomes by combining clinical and histological data. Our study is the first to externally validate JHGC in a non-Japanese IgAN population. While JHGC can be applied to our population, we have significantly increased the number of cases in the group with eGFR ≥ 60 mL/min/1.732 m^2^ and UPCR < 0.5 g/day. Based on our findings, we propose the adoption of a new classification specifically for Taiwanese IgAN patients.

## Figures and Tables

**Table 1:** Baseline characteristics according to Japan classification system (based on merged Japan histology and clinical classifications).

Risk Classification	Total (n = 359)	Low (n = 64)	Moderate (n = 113)	High (n = 79)	Very High (n = 103)	*p*
Variables	Median (Q1, Q3) for continuous variables; n (%) for categorical variables					
Demographic data						
Age (years old)	46 (37, 59)	46 (34.5, 59)	43 (32, 55)	50 (36, 63)	51 (42, 60)	<0.001
Body mass index (kg/m^2^)	23.47 (20.70, 27.45)	23.07 (20.96, 27.22)	23.96 (20.69, 27.75)	23.44 (20.70, 27.47)	22.60 (20.17, 26.07)	0.901
Male	188 (52.4%)	34 (53.1%)	56 (49.6%)	45 (57%)	53 (51.5%)	0.784
Blood data						
Blood WBC (/cumm)	7630 (6100, 9230)	6770 (5630, 8290)	7870 (6100, 9220)	7320 (6100, 9880)	7820 (6390, 9390)	0.26
Hemoglobin (g/dL)	12.7 (10.9, 14.1)	13.1 (12, 14.5)	13.3 (12.1, 14.6)	12.5 (10.5, 14.1)	11.2 (9.8, 13.1)	<0.001
Platelet (×10^3^/cumm)	247 (202, 293)	239 (202, 292.5)	252 (215, 324)	249 (196, 286)	238 (196, 277)	0.001
Fasting glucose (mg/dL)	94 (86, 107)	93 (85, 99)	94 (87, 108)	95.5 (86, 105)	95 (86, 110)	0.209
Glycated hemoglobin (%)	5.5 (5.3, 5.8)	5.5 (5.2, 5.8)	5.5 (5.3, 5.8)	5.6 (5.4, 6)	5.6 (5.2, 5.9)	0.4
Albumin (g/dL)	4 (3.6, 4.2)	4.2 (4, 4.5)	4 (3.7, 4.3)	3.8 (3.4, 4)	3.7 (3.5, 4)	<0.001
Total cholesterol (mg/dL)	187.5 (164, 219)	172.5 (154, 191)	191 (165, 219)	186 (162, 221)	196.5 (170, 228)	0.07
Triglyceride (mg/dL)	122 (83.5, 184.5)	102 (66, 138)	118 (84, 185)	126.5 (87, 180)	143 (95, 195)	0.003
Low-density lipoprotein (mg/dL)	110.5 (90, 142)	99 (85, 122)	108.5 (90, 141)	110.5 (91, 140)	123.5 (92, 147)	0.095
High-density lipoprotein (mg/dL)	47.4 (31.2, 64)	47.4 (35.8, 59.8)	48.8 (34.2, 66)	47.3 (31.7, 69)	42.4 (29, 64.4)	0.379
Blood urea nitrogen (mg/dL)	20 (14, 31)	15 (13, 22)	15 (12, 19)	22 (17, 29)	38 (26, 50)	<0.001
Creatinine (mg/dL)	1.3 (0.9, 2.1)	1 (0.7, 1.4)	1 (0.7, 1.2)	1.5 (1.2, 1.8)	2.7 (1.8, 4.2)	<0.001
Estimated glomerular filtrate rate (min/min.1.732 m^2^)	54.04 (32.18, 86.29)	84.22 (48.1, 106.59)	83.06 (66.81, 101.4)	47.2 (37.96, 69.39)	25.08 (14.97, 36.9)	<0.001
Uric acid (mg/dL)	6.6 (5.3, 8.1)	6.3 (5, 7.3)	6 (4.9, 7)	7 (5.9, 8.1)	7.5 (6.3, 9.1)	<0.001
C3 (mg/dL)	107.8 (93.3, 120.9)	110.2 (95.9, 130.2)	114.5 (98.6, 124.7)	105 (94.9, 118.6)	97.4 (78.2, 118.1)	0.008
C4 (mg/dL)	31 (24, 37)	28 (22, 36)	29 (24, 35)	31 (24, 36)	34 (28, 41)	<0.001
IgG (mg/dL)	1061 (899, 1266)	1184 (1016, 1298)	1065 (908, 1266)	1037 (730, 1251)	1012 (823, 1231)	0.072
IgA (mg/dL)	328 (256, 418)	318 (258, 399)	336 (257, 441)	323 (251, 422)	329 (252, 393)	0.112
IgM (mg/dL)	98 (72, 132)	112 (86, 138)	107 (80, 138)	96 (66, 127)	88 (57, 126)	<0.001
IgE (mg/dL)	69.3 (13.6, 95.3)	95.3 (30.4, 654)	69.9 (69.3, 70.5)	13.6 (13.6, 13.6)	4.7 (4.7, 4.7)	0.258
Urinary data						
Urine protein–creatinine ratio (mg/g)	1154.5 (420.6, 2612.09)	190 (96.94, 320)	1060 (520, 2020)	1431.5 (684.41, 3240.12)	2070 (1237.64, 4464)	<0.001
Hematuria	310 (86.4%)	55 (85.9%)	93 (82.3%)	71 (89.9%)	91 (88.3%)	0.448
Pyuria	205 (57.1%)	31 (48.4%)	72 (63.7%)	43 (54.4%)	59 (57.3%)	0.241
Pathological data based on Oxford classification						
M	205 (58.4%)	17 (27.4%)	62 (54.9%)	52 (67.5%)	74 (74.7%)	<0.001
E	104 (29.6%)	5 (8.1%)	26 (23%)	27 (35.1%)	46 (46.5%)	<0.001
S	219 (62.4%)	11 (17.7%)	70 (61.9%)	53 (68.8%)	85 (85.9%)	<0.001
T1	72 (20.5%)	1 (1.6%)	6 (5.3%)	18 (23.4%)	47 (47.5%)	<0.001
T2	31 (8.8%)	1 (1.6%)	0 (0%)	4 (5.2%)	26 (26.3%)	<0.001
C1	38 (10.7%)	0 (0%)	13 (11.5%)	13 (16.7%)	12 (11.9%)	0.046
C2	11 (3.1%)	0 (0%)	1 (0.9%)	3 (3.8%)	7 (6.9%)	0.123

Oxford MEST-C, derived from pathology, encompassing mesangial hypercellularity (M), endocapillary hypercellularity (E), segmental glomerulosclerosis (S), tubular atrophy/interstitial fibrosis (T), and crescents (C). A score of T0, T1, or T2 is given if the percentage of involved cortical area is 0 to 25, 26 to 50, or >50 percent, respectively. This feature is defined as present if cellular and/or fibrocellular crescents are present in at least one glomerulus (C1), present in at least 25 percent of glomeruli (C2), or absent (C0).

**Table 2:** Baseline characteristics according to new classification system (based on new merged histology and new clinical classifications).

Risk Classification	Total (n = 359)	Very Low (n = 54)	Low (n = 110)	Moderate (n = 59)	High (n = 24)	Very High (n = 112)	*p*
Variables	Median (Q1, Q3) for continuous variables; n (%) for categorical variables						
Demographic data							
Age (years old)	46 (37, 59)	42.5 (32, 53)	44 (32, 58)	49 (40, 60)	59.5 (36.5, 66.5)	51 (42, 60)	<0.001
Body mass index (kg/m^2^)	23.47 (20.70, 27.45)	23.33 (20.87, 27.29)	23.50 (20.69, 27.85)	23.35 (20.70, 27.47)	23.92 (20.31, 27.17)	22.78 (21.01, 27.01)	0.966
Male	188 (52.4%)	31 (57.4%)	52 (47.3%)	33 (55.9%)	14 (58.3%)	58 (51.8%)	0.679
Blood data							
Blood white blood cell (/cumm)	7630 (6100, 9230)	7300 (5800, 8400)	7720 (5840, 8965)	7735 (6330, 9810)	7170 (6010, 9810)	7700 (6365, 9490)	0.23
Hemoglobin (g/dL)	12.7 (10.9, 14.1)	13.6 (12.4, 14.7)	13.1 (11.9, 14.4)	12.6 (11, 14.2)	12.1 (10.1, 13.2)	11.3 (9.8, 13.1)	<0.001
Platelet (×10^3^/cumm)	247 (202, 293)	249 (203, 313)	250 (203, 310)	249 (204, 286)	240.5 (189, 263)	236 (195.5, 278.5)	0.155
Fasting glucose (mg/dL)	94 (86, 107)	88.5 (81.5, 98.5)	95 (87, 108)	93 (86, 102)	98 (84, 105.5)	95.5 (86.5, 110)	0.1
Glycated hemoglobin (%)	5.5 (5.3, 5.8)	5.4 (5.3, 5.8)	5.5 (5.3, 5.8)	5.6 (5.4, 5.9)	5.5 (5.3, 6.1)	5.6 (5.2, 5.8)	0.525
Albumin (g/dL)	4 (3.6, 4.2)	4.3 (4, 4.5)	4 (3.7, 4.3)	4 (3.5, 4.2)	3.8 (3.5, 4.2)	3.8 (3.5, 4)	<0.001
Total cholesterol (mg/dL)	187.5 (164, 219)	175 (154, 208)	186 (164.5, 215.5)	193.5 (167, 217)	191.5 (167, 225)	195 (164, 225)	0.254
Triglyceride (mg/dL)	122 (83.5, 184.5)	103 (67, 140)	113 (75, 186)	121.5 (92, 192)	124 (78.5, 166)	145.5 (99, 192)	0.008
Low-density lipoprotein (mg/dL)	110.5 (90, 142)	99 (85, 133)	105.5 (91, 141)	110 (90, 139)	109 (95.5, 129)	120 (91, 147)	0.438
High-density lipoprotein (mg/dL)	47.4 (31.2, 64)	52.2 (36.2, 61.6)	47.6 (30.2, 66)	45.4 (33.4, 68.8)	53.7 (46.6, 72.2)	40.4 (28.6, 63.8)	0.259
Blood urea nitrogen (mg/dL)	20 (14, 31)	14 (12, 17)	15.5 (13, 20)	21 (16, 29)	25.5 (19, 31.5)	37 (26, 50)	<0.001
Creatinine (mg/dL)	1.3 (0.9, 2.1)	0.9 (0.7, 1)	1 (0.7, 1.2)	1.5 (1.1, 1.7)	1.6 (1.4, 2.4)	2.6 (1.8, 4.6)	<0.001
Estimated glomerular filtrate rate (min/min.1.732 m^2^)	54.04 (32.18, 86.29)	93.91 (79.59, 111.48)	80.76 (60.73, 101.4)	49.36 (40.37, 70.7)	42.54 (29.27, 55.27)	26.25 (14.84, 37.54)	<0.001
Uric acid (mg/dL)	6.6 (5.3, 8.1)	6.1 (5, 7.2)	6.1 (4.9, 7.2)	6.5 (5.8, 7.6)	7.7 (5.7, 9.6)	7.5 (6.2, 9)	<0.001
C3 (mg/dL)	107.8 (93.3, 120.9)	113.9 (101.1, 130.2)	108.3 (95.4, 122.9)	105.9 (90.8, 118.6)	105.3 (94.9, 112.7)	97.9 (78.9, 120)	0.077
C4 (mg/dL)	31 (24, 37)	31 (22, 38)	28 (23, 33)	32 (25, 35)	30 (23, 37)	34 (28, 41)	<0.001
IgG (mg/dL)	1061 (899, 1266)	1166 (999, 1303)	1068 (920, 1253)	1079 (820, 1327)	1036 (730, 1251)	1007 (807, 1231)	0.119
IgA (mg/dL)	328 (256, 418)	329 (257, 431)	334 (260, 441)	327 (234, 427)	314 (261, 354)	327 (250, 393)	0.649
IgM (mg/dL)	98 (72, 132)	117 (88, 141)	108 (84, 138)	96 (59, 127)	93 (66, 115)	87 (57, 121)	0.001
IgE (mg/dL)	69.3 (13.6, 95.3)	82.3 (49.8, 374.7)	70.5 (70.5, 70.5)	13.6 (13.6, 13.6)	-	4.7 (4.7, 4.7)	0.277
Urinary data							
Urine protein–creatinine ratio (mg/g)	1154.5 (420.6, 2612.09)	240 (140, 381.86)	1116.47 (530, 2150)	1330 (550, 2749.92)	1160 (547.23, 3580)	1937 (1080, 3852)	<0.001
Hematuria	310 (86.4%)	48 (88.9%)	89 (80.9%)	51 (86.4%)	22 (91.7%)	100 (89.3%)	0.408
Pyuria	205 (57.1%)	22 (40.7%)	72 (65.5%)	31 (52.5%)	16 (66.7%)	64 (57.1%)	0.035
Pathological data based on Oxford classification							
M	205 (58.4%)	20 (37%)	50 (46.3%)	41 (69.5%)	14 (63.6%)	80 (74.0%)	<0.001
E	104 (29.6%)	6 (11.1%)	24 (22.2%)	19 (32.2%)	7 (31.8%)	48 (44.4%)	<0.001
S	219 (62.4%)	20 (37%)	54 (50%)	44 (74.6%)	11 (50%)	90 (83.3%)	<0.001
T1	72 (20.5%)	0 (0%)	2 (1.9%)	18 (30.5%)	3 (13.6%)	49 (45.4%)	<0.001
T2	31 (8.8%)	0 (0%)	1 (0.9%)	2 (3.4%)	0 (0%)	28 (25.9%)	<0.001
C1	38 (10.7%)	2 (3.7%)	9 (8.3%)	11 (18.6%)	4 (17.4%)	12 (10.9%)	0.069
C2	11 (3.1%)	0 (0%)	1 (0.9%)	3 (5.1%)	0 (0%)	7 (6.4%)	0.078

## Data Availability

No new data were created or analyzed in this study. Data sharing is not applicable to this article.

## Supplementary Materials

The following supporting information can be downloaded at: https://www.mdpi.com/article/10.3390/jcm12237339/s1, Figure S1: Process of Taiwanese IgAN in Japanese Histological Grade Classification (JHGC); Table S1: Baseline characteristics according to Japan classification system (based on Japan histological classifications) (4H); Table S2: Baseline characteristics according to Japan classification system (based on Japan clinical classifications) (3C); Table S3: Baseline characteristics according to new classification system (based new clinical classifications) (new 4C).
