# Supplementary material for: The Feasibility of Japanese Histological Grade Classification for Predicting Renal Function Deterioration among Taiwanese Individuals with IgA Nephropathy
Source: J Clin Med. 2023 Nov 27;12(23):7339. doi: 10.3390/jcm12237339 (PMC10707328; doi:10.3390/jcm12237339)
Supplement: Supplementary file 1 [file jcm-12-07339-s001.zip › jcm-2707320-supplementary.pdf]

Supplementary data

Table S1. Baseline characteristics according to Japan classification system (based **on Japan histological** classifications) (4H)

Risk classification	Total(n=359)	Grade I (n=143)	Grade II (n=80)	Grade III (n=69)	Grade IV (n=67)	*p*
Variables	median (Q1, Q3) for continuous variables; n (%) for categorical variables					
Demographic data						
Age (years old)	46 (37, 59)	44 (31, 59)	48.5 (41.5, 58)	55 (42, 61)	45 (38, 56)	0.02
Body mass index (kg/m^2)	23.47 (20.70, 27.45)	23.07 (20.76, 27.17)	24.48 (20.43, 27.85)	24.25 (21.83, 27.58)	21.98 (19.92, 26.23)	0.349
Male	188 (52.4%)	73 (51%)	43 (53.8%)	40 (58%)	32 (47.8%)	0.663
Blood data						
Blood WBC (/cumm)	7630 (6100, 9230)	7300 (5840, 8400)	7980 (6070, 9780)	7630 (6300, 9390)	8335 (6800, 10000)	0.04
Hemoglobin (g/dl)	12.7 (10.9, 14.1)	13.1 (11.9, 14.3)	12.9 (11, 14.3)	12.5 (11.1, 13.7)	10.9 (9.1, 13.1)	<0.001
Platelet (*10^3/cumm)	247 (202, 293)	247 (203, 314)	240.5 (198.5, 285.5)	252 (212, 290)	226 (195, 261)	0.237
Fasting glucose (mg/dl)	94 (86, 107)	94 (86, 104)	94 (86, 108)	96 (87, 111)	92 (82, 103)	0.71
Glycated hemoglobin (%)	5.5 (5.3, 5.8)	5.5 (5.3, 5.8)	5.5 (5.3, 5.8)	5.7 (5.3, 6)	5.5 (5.1, 5.7)	0.092
Albumin (g/dl)	4 (3.6, 4.2)	4 (3.8, 4.3)	4 (3.7, 4.3)	3.8 (3.5, 4)	3.7 (3.5, 4)	<0.001
Total cholesterol (mg/dl)	187.5 (164, 219)	180 (163, 214)	191 (167, 214)	195.5 (171.5, 223)	195 (159, 231)	0.299
Triglyceride (mg/dl)	122 (83.5, 184.5)	104 (72, 150)	119 (96, 186)	144 (83, 199)	147 (96, 191)	0.002
Low-density lipoprotein (mg/dl)	110.5 (90, 142)	103 (89.5, 139.5)	110 (85, 134)	117 (93.5, 144)	122 (92, 147)	0.206
High-density lipoprotein (mg/dl)	47.4 (31.2, 64)	48.9 (34.6, 63)	50.8 (35.2, 67)	41 (28, 67)	39.4 (29.6, 59.8)	<0.001
Blood urea nitrogen (mg/dl)	20 (14, 31)	15 (13, 22)	18 (15, 24)	25 (18, 38)	40 (28, 61)	<0.001
Creatinine (mg/dl)	1.3 (0.9, 2.1)	1 (0.7, 1.4)	1.3 (1, 1.6)	1.7 (1.1, 2.5)	3.2 (1.9, 6.9)	<0.001

Estimated glomerular filtrate rate (min/min.1.732m^2)	54.04 (32.18, 86.29)	81.8 (54.42, 105.47)	60.25 (42.46, 80.65)	41.37 (26.93, 67.74)	20.97 (7.91, 34.22)	<0.001
Uric acid (mg/dl)	6.6 (5.3, 8.1)	6.3 (5, 7.4)	6.4 (5.3, 7.7)	7.2 (5.9, 8.4)	7.5 (6.2, 9.3)	<0.001
C3 (mg/dl)	112 (97, 127)	113 (99, 130)	112 (97, 124)	110 (99, 126)	112 (90, 124)	0.316
C4 (mg/dl)	31 (24, 37)	28 (22, 35)	30 (25, 35)	32 (25, 38)	35 (27, 41)	<0.001
IgG (mg/dl)	1061 (899, 1266)	1155 (951, 1281)	1062 (900, 1263)	990 (780, 1183)	1041 (795, 1287)	0.054
IgA (mg/dl)	328 (256, 418)	326 (261, 418)	345 (256, 443)	337 (256, 410)	309 (248, 369)	0.391
IgM (mg/dl)	98 (72, 132)	107 (86, 139)	102 (72, 119)	89 (63, 140)	87 (57, 121)	0.001
IgE (mg/dl)	69.3 (13.6, 95.3)	82.9 (50.5, 374.7)	41.4 (13.6, 69.3)	-	4.7 (4.7, 4.7)	0.158
Urinary data						
Urine protein-creatinine-ratio (mg/g)	1154.5 (420.6, 2612.09)	543.62 (200, 1510)	1179.99 (401.26, 2470)	1482.43 (882.61, 2785.45)	2647.9 (1319, 4660)	<0.001
Hematuria	310 (86.4%)	119 (83.2%)	70 (87.5%)	60 (87%)	61 (91%)	0.495
Pyuria	205 (57.1%)	85 (59.4%)	45 (56.3%)	36 (52.2%)	39 (58.2%)	0.782
Pathological data based on Oxford classification						
M	205 (58.4%)	57 (41%)	51 (63.7%)	50 (74.6%)	47 (72.3%)	<0.001
E	104 (29.6%)	26 (18.7%)	22 (27.5%)	26 (38.8%)	30 (46.2%)	<0.001
S	219 (62.4%)	47 (33.8%)	60 (75%)	57 (85.1%)	55 (84.6%)	<0.001
T1	72 (20.5%)	4 (2.9%)	13 (16.3%)	25 (37.3%)	30 (46.2%)	<0.001
T2	31 (8.8%)	1 (0.7%)	1 (1.3%)	6 (9%)	23 (35.4%)	<0.001
C1	72 (20.5%)	4 (2.9%)	13 (16.3%)	25 (37.3%)	30 (46.2%)	<0.001

C2	31 (8.8%)	1 (0.7%)	1 (1.3%)	6 (9%)	23 (35.4%)	<0.001

Table S2. Baseline characteristics according to Japan classification system (based **on Japan clinical** classifications) (3C)

Risk classification	Total(n=359)	Grade I (n=103)	Grade II (n=103)	Grade III (n=149)	*p* value
Variables	median (Q1, Q3) for continuous variables; n (%) for categorical variables				
Demographic data					
Age (years old)	46 (37, 59)	47 (39, 59)	41 (29, 51)	53 (42, 62)	<0.001
Body mass index (kg/m^2)	23.47 (20.70, 27.45)	23.92 (21.05, 27.54)	23.494 (20.74, 27.88)	22.60 (20.27, 26.23)	0.277
Male	188 (52.4%)	56 (54.4%)	53 (49.5%)	79 (53%)	0.567
Blood data					
Blood WBC (/cumm)	7630 (6100, 9230)	7360 (5630, 8900)	7880 (6200, 9260)	7440 (6300, 9590)	0.23
Hemoglobin (g/dl)	12.7 (10.9, 14.1)	13.1 (11.8, 14.3)	13.3 (12.4, 14.7)	11.3 (9.9, 13.1)	<0.001
Platelet (*10^3/cumm)	247 (202, 293)	234 (200, 292)	267 (231, 328)	237 (195, 277)	<0.001
Fasting glucose (mg/dl)	94 (86, 107)	93 (85, 100)	93 (87, 107)	96 (86, 109.5)	0.272
Glycated hemoglobin (%)	5.5 (5.3, 5.8)	5.5 (5.3, 5.8)	5.5 (5.3, 5.9)	5.5 (5.3, 5.8)	0.816
Albumin (g/dl)	4 (3.6, 4.2)	4.2 (4, 4.4)	3.9 (3.6, 4.2)	3.8 (3.4, 4)	<0.001
Total cholesterol (mg/dl)	187.5 (164, 219)	175 (157, 196)	194 (165, 228)	196 (168, 226)	0.001
Triglyceride (mg/dl)	122 (83.5, 184.5)	113.5 (77, 169)	115.5 (76, 191)	133.5 (91.5, 189.5)	0.086
Low-density lipoprotein (mg/dl)	110.5 (90, 142)	101 (85, 125)	111.5 (93.5, 146)	120 (91, 146)	0.01
High-density lipoprotein (mg/dl)	47.4 (31.2, 64)	47.4 (34, 57.8)	47.4 (30.2, 68)	47.5 (31.3, 67.6)	0.72
Blood urea nitrogen (mg/dl)	20 (14, 31)	17 (13, 25)	15 (12, 18)	31 (23,	<0.00

				44)	1
Creatinine (mg/dl)	1.3 (0.9, 2.1)	1.2 (0.8, 1.6)	0.9 (0.7, 1.1)	2.1 (1.6, 3.3)	<0.001
Estimated glomerular filtrate rate (min/min.1.732m^2)	54.04 (32.18, 86.29)	66.41 (44.64, 96.2)	85.88 (71.15, 104.63)	31.02 (18.16, 42.23)	<0.001
Uric acid (mg/dl)	6.6 (5.3, 8.1)	6.3 (5.1, 7.6)	6 (4.9, 7)	7.4 (6.2, 9.1)	<0.001
C3 (mg/dl)	112 (97, 127)	110 (98, 125)	118 (102, 134)	107 (92, 121)	0.001
C4 (mg/dl)	31 (24, 37)	30 (23, 37)	29 (24, 33)	33 (26, 39)	0.004
IgG (mg/dl)	1061 (899, 1266)	1168 (994, 1299)	1036 (856, 1243)	1042 (822, 1245)	0.02
IgA (mg/dl)	328 (256, 418)	316 (253, 393)	338 (260, 443)	330 (254, 410)	0.109
IgM (mg/dl)	98 (72, 132)	96 (74, 131)	108 (85, 140)	89 (59, 127)	0.012
IgE (mg/dl)	69.3 (13.6, 95.3)	82.3 (49.8, 374.7)	70.5 (70.5, 70.5)	9.2 (4.7, 13.6)	0.153
Urinary data					
Urine protein-creatinine-ratio (mg/g)	1154.5 (420.6, 2612.09)	235.87 (140, 371.49)	1510 (970, 2620)	1934 (1107.12, 3970)	<0.001
Hematuria	310 (86.4%)	90 (87.4%)	88 (82.2%)	132 (88.6%)	0.33
Pyuria	205 (57.1%)	53 (51.5%)	66 (61.7%)	86 (57.7%)	0.327
Pathological data based on Oxford classification					
M	205 (58.4%)	42 (41.6%)	60 (56.1%)	103(72.0%)	<0.001
E	104 (29.6%)	10 (9.9%)	31 (29%)	63 (44.1%)	<0.001
S	219 (62.4%)	37 (36.6%)	71 (66.4%)	111 (77.6%)	<0.001
T1	72 (20.5%)	8 (7.9%)	7 (6.5%)	57 (39.9%)	<0.001

T2	31 (8.8%)	3 (3%)	1 (0.9%)	27 (18.9%)	<0.001
C1	38 (10.7%)	5 (4.9%)	14 (13.1%)	19 (13%)	0.067
C2	11 (3.1%)	0 (0%)	2 (1.9%)	9 (6.2%)	0.014

Table S3. Baseline characteristics according to new classification system (based **new clinical** classifications) (new 4C)

Risk classification	Total(n=359)	Grade I (n=58)	Grade II (n=107)	Grade III (n=45)	Grade IV (n=149)	*P value*
Variables	median (Q1, Q3) for continuous variables; n (%) for categorical variables					
Demographic data						
Age (years old)	46 (37, 59)	43 (32, 53)	41 (29, 51)	56 (46, 62)	53 (42, 62)	<0.001
Body mass index (kg/m^2)	23.47 (20.70, 27.45)	23.33 (20.96, 27.04)	23.49 (20.74, 27.88)	26.50 (21.05, 27.93)	22.60 (20.27, 26.23)	0.275
Male	188 (52.4%)	32 (55.2%)	53 (49.05%)	24 (53.3%)	79 (53%)	0.91
Blood data						
Blood WBC (/cumm)	7630 (6100, 9230)	7300 (5800, 8700)	7880 (6200, 9260)	7420 (5620, 9230)	7440 (6300, 9590)	0.375
Hemoglobin (g/dl)	12.7 (10.9, 14.1)	13.5 (12.4, 14.6)	13.3 (12.4, 14.7)	12.2 (10.5, 13.1)	11.3 (9.9, 13.1)	<0.001
Platelet (*10^3/cumm)	247 (202, 293)	253 (203, 307)	267 (231, 328)	212 (184, 247)	237 (195, 277)	<0.001
Fasting glucose (mg/dl)	94 (86, 107)	88.5 (81, 99)	93 (87, 107)	95 (89, 108)	96 (86, 109.5)	0.049
Glycated hemoglobin (%)	5.5 (5.3, 5.8)	5.4 (5.3, 5.8)	5.5 (5.3, 5.9)	5.6 (5.3, 5.9)	5.5 (5.3, 5.8)	0.594
Albumin (g/dl)	4 (3.6, 4.2)	4.3 (4, 4.5)	3.9 (3.6, 4.2)	4.2 (3.9, 4.3)	3.8 (3.4, 4)	<0.001
Total cholesterol (mg/dl)	187.5 (164, 219)	180 (154, 208)	194 (165, 228)	172 (162, 191.5)	196 (168, 226)	0.001
Triglyceride (mg/dl)	122 (83.5, 184.5)	103 (66, 141)	115.5 (76, 191)	129 (93, 180)	133.5 (91.5, 189.5)	0.031
Low-density lipoprotein (mg/dl)	110.5 (90, 142)	101 (86, 132)	111.5 (93.5, 146)	100 (83, 119.5)	120 (91, 146)	0.019
High-density lipoprotein (mg/dl)	47.4 (31.2, 64)	52.4 (36.2, 61.8)	47.4 (30.2, 68)	39.8 (32.3, 53.8)	47.5 (31.3, 67.6)	0.27
Blood urea nitrogen (mg/dl)	20 (14, 31)	14 (12, 17)	15 (12, 18)	26 (19, 36)	31 (23, 44)	<0.001
Creatinine (mg/dl)	1.3 (0.9, 2.1)	0.9 (0.7, 1)	0.9 (0.7, 1.1)	1.6 (1.4, 2.1)	2.1 (1.6, 3.3)	<0.001
Estimated glomerular filtrate rate	54.04 (32.18,	92.94 (79.59,	85.88 (71.15,	42.42 (32.98,	31.02 (18.16,	<0.0

(min/min.1.732m^2)	86.29)	111.21)	104.63)	48.04)	42.23)	01
Uric acid (mg/dl)	6.6 (5.3, 8.1)	6.1 (5, 7.2)	6 (4.9, 7)	6.8 (5.8, 7.9)	7.4 (6.2, 9.1)	<0.001
C3 (mg/dl)	112 (97, 127)	112 (105, 129)	118 (102, 134)	107 (94, 115)	107 (92, 121)	<0.001
C4 (mg/dl)	31 (24, 37)	31 (22, 38)	29 (24, 33)	30 (25, 37)	33 (26, 39)	0.01
IgG (mg/dl)	1061 (899, 1266)	1166 (999, 1303)	1036 (856, 1243)	1179 (973, 1263)	1042 (822, 1245)	0.044
IgA (mg/dl)	328 (256, 418)	329 (259, 425)	338 (260, 443)	289 (195, 332)	330 (254, 410)	0.024
IgM (mg/dl)	98 (72, 132)	117 (88, 152)	108 (85, 140)	79 (59, 110)	89 (59, 127)	<0.001
IgE (mg/dl)	69.3 (13.6, 95.3)	82.3 (49.8, 374.7)	70.5 (70.5, 70.5)	-	9.2 (4.7, 13.6)	0.153
Urine protein-creatinine-ratio (mg/g)	1154.5 (420.6, 2612.09)	260 (140, 390)	1510 (970, 2620)	233.19 (120, 320)	1934 (1107.12, 3970)	<0.001
Hematuria	310 (86.4%)	51 (87.9%)	88 (82.2%)	39 (86.7%)	132 (88.6%)	0.527
Pyuria	205 (57.1%)	25 (43.1%)	66 (61.7%)	28 (62.2%)	86 (57.7%)	0.112
Pathological data based on Oxford classification						
M	205 (58.4%)	22 (37.9%)	60 (56.1%)	20 (46.5%)	103 (72.0%)	<0.001
E	104 (29.6%)	6 (10.3%)	31 (29%)	4 (9.3%)	63 (44.1%)	<0.001
S	219 (62.4%)	23 (39.7%)	71 (66.4%)	14 (32.6%)	111 (77.6%)	<0.001
T1	72 (20.5%)	0 (0%)	7 (6.5%)	8 (18.6%)	57 (39.9%)	<0.001
T2	31 (8.8%)	0 (0%)	1 (0.9%)	3 (7%)	27 (18.9%)	<0.001
C1	38 (10.7%)	3 (5.2%)	14 (13.1%)	2 (4.5%)	19 (13%)	0.179
C2	11 (3.1%)	0 (0%)	2 (1.9%)	0 (0%)	9 (6.2%)	0.065

Figure S1. Process of Taiwanese IgAN in Japanese Histological Grade Classification (JHGC)

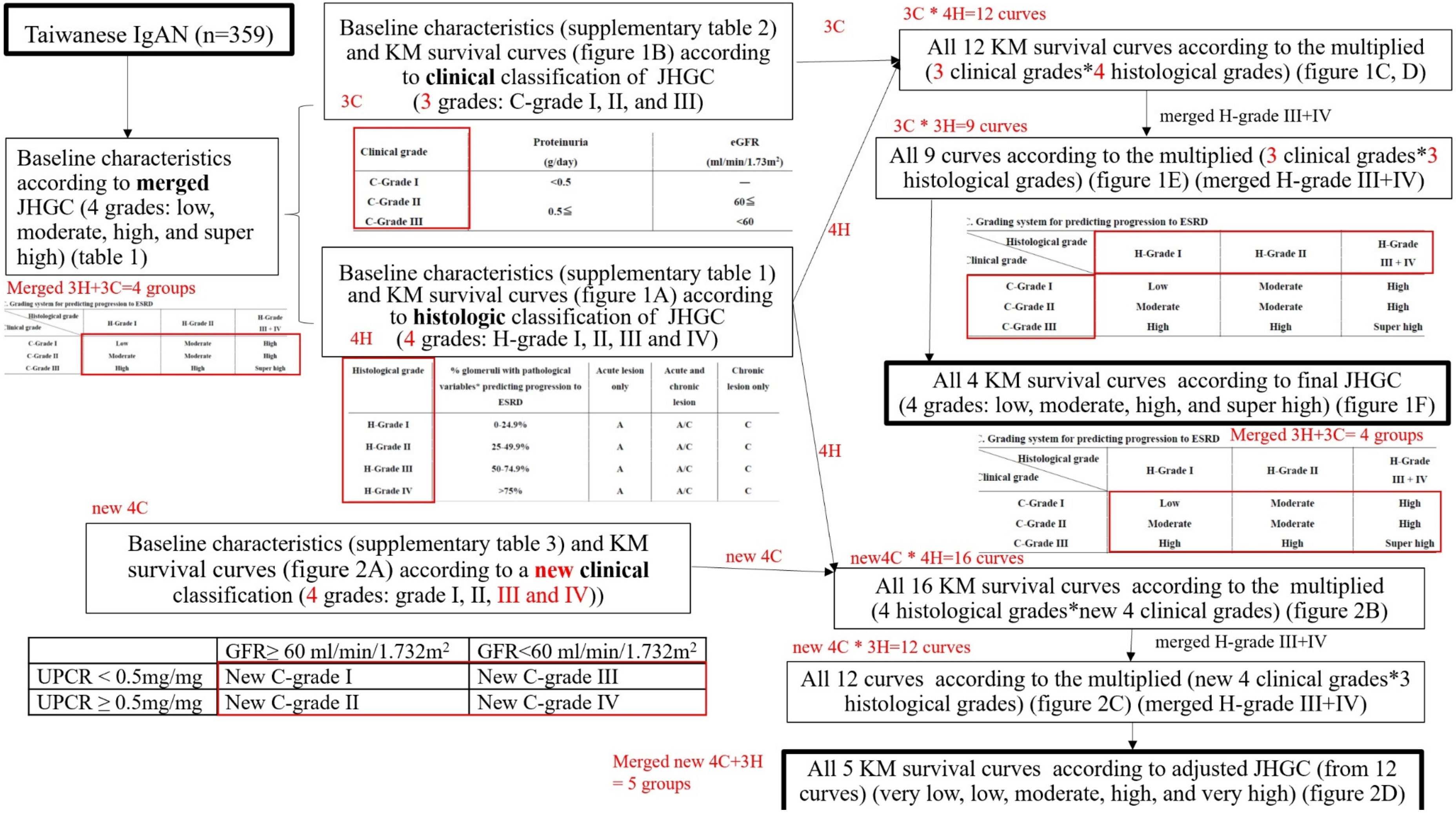

- body mass index (BMI)
- blood urea nitrogen (BUN)
- confidence intervals (CI)
- complement 3 (C3)
- end-stage kidney disease (ESKD)
- fasting blood sugar (FBS)
- glomerular filtration rate (GFR)
- glomerular lesion percentage score (GLPS)
- glycated hemoglobin (HbA1c)
- hazard risk (HR)
- high-density lipoprotein (HDL)
- Immunoglobulin A nephropathy (IgAN)
- Japanese Histological Grade Classification (JHGC)
- low-density lipoprotein (LDL)
- Mesangial hypercellularity, endocapillary hypercellularity, segmental glomerulosclerosis, tubular atrophy/interstitial fibrosis, and crescents (MEST-C)
- serum creatinine (SCr)
- total cholesterol (TC)
- uric acid (UA) level
- urine protein-creatinine ratio (mg/mg) (UPCR)
